# The Position of His-Tag in Recombinant OspC and Application of Various Adjuvants Affects the Intensity and Quality of Specific Antibody Response after Immunization of Experimental Mice

**DOI:** 10.1371/journal.pone.0148497

**Published:** 2016-02-05

**Authors:** Michal Krupka, Josef Masek, Lucia Barkocziova, Pavlina Turanek Knotigova, Pavel Kulich, Jana Plockova, Robert Lukac, Eliska Bartheldyova, Stepan Koudelka, Radka Chaloupkova, Marek Sebela, Daniel Zyka, Ladislav Droz, Roman Effenberg, Miroslav Ledvina, Andrew D. Miller, Jaroslav Turanek, Milan Raska

**Affiliations:** 1 Department of Immunology, Faculty of Medicine and Dentistry, Palacky University Olomouc, Olomouc, Czech Republic; 2 Department of Pharmacology and Immunotherapy, Veterinary Research Institute, Brno, Czech Republic; 3 International Clinical Research Center, St. Anne´s University Hospital, Brno, Czech Republic; 4 Loschmidt Laboratories, Department of Experimental Biology and Research Centre for Toxic Compounds in the Environment RECETOX, Masaryk University, Brno, Czech Republic; 5 Centre of the Region Hana for Biotechnological and Agricultural Research, Faculty of Science, Palacky University Olomouc, Olomouc, Czech Republic; 6 Apigenex, Prague, Czech Republic; 7 Department of Chemistry of Natural Compounds University of Chemistry and Technology, Prague, Czech Republic; 8 King's College London, Institute of Pharmaceutical Science, London, United Kingdom, and GlobalAcorn Ltd, London, United Kingdom; University of Kentucky College of Medicine, UNITED STATES

## Abstract

Lyme disease, *Borrelia burgdorferi*-caused infection, if not recognized and appropriately treated by antibiotics, may lead to chronic complications, thus stressing the need for protective vaccine development. The immune protection is mediated by phagocytic cells and by *Borrelia*-specific complement-activating antibodies, associated with the Th1 immune response. Surface antigen OspC is involved in *Borrelia* spreading through the host body. Previously we reported that recombinant histidine tagged (His-tag) OspC (rOspC) could be attached onto liposome surfaces by metallochelation. Here we report that levels of OspC-specific antibodies vary substantially depending upon whether rOspC possesses an N' or C' terminal His-tag. This is the case in mice immunized: (a) with rOspC proteoliposomes containing adjuvants MPLA or non-pyrogenic MDP analogue MT06; (b) with free rOspC and Montanide PET GEL A; (c) with free rOspC and alum; or (d) with adjuvant-free rOspC. Stronger responses are noted with all N'-terminal His-tag rOspC formulations. OspC-specific Th1-type antibodies predominate post-immunization with rOspC proteoliposomes formulated with MPLA or MT06 adjuvants. Further analyses confirmed that the structural features of soluble N' and C' terminal His-tag rOspC and respective rOspC proteoliposomes are similar including their thermal stabilities at physiological temperatures. On the other hand, a change in the position of the rOspC His-tag from N' to C' terminal appears to affect substantially the immunogenicity of rOspC arguably due to steric hindrance of OspC epitopes by the C' terminal His-tag itself and not due to differences in overall conformations induced by changes in the His-tag position in rOspC variants.

## Introduction

Spirochete *Borrelia burgdorferi* s. l. is the causative agent of Lyme disease. Although at least nine *Borrelia* species are currently considered potentially pathogenic [1] *B*. *afzelii*, *B*. *garinii*, and *B*. *burgdorferi* s. s. still predominates in Europe and *B*. *burgdorferi* s. s. in the USA. If untreated by antibiotic therapy in time, Lyme disease can develop into a chronic phase of infection with long-lasting neural, cardiovascular, cutaneous, or orthopedic complications [2, 3]. In some patients, the chronic phase can develop in spite of intensive antibiotic treatment. This complication emphasizes the need for the development of protective vaccines to control either *Borrelia* transmission from tick to vertebrate host or subsequently from spreading through the mammalian host organism [4–6]. Currently, (Outer surface protein) OspA and OspC are considered as the most promising antigens for vaccination purposes [5].

OspC is a lipoprotein antigen of approximately 23 kDa and 210 amino acid residues localized on the *Borrelia* surface. The expression of OspC is required for *Borrelia* transmission from tick to vertebrate host and for the initial stage of vertebrate host infection. *Borrelia* lacking OspC and regulatory sigma factors RpoS, RpoN, or Rrp2 are severely impaired in their pathogenicity [7, 8]. The native conformation of OspC seems to be crucial for the induction of borreliacidal antibodies because if this antigen is denatured, the induction of the antibodies fails [9]. Epitopes recognized by protective antibodies were mapped to the C-terminal regions of OspC, specifically the regions loop 5, helix 5, and conserved last 20 amino acid residues [10, 11].

Nascent OspC contains a N-terminal lipidation signal sequence leading to the modification of OspC by addition of a the hydrophobic triacylglycerol moiety important for the subsequent integration of OspC into the outer membrane of *Borrelia* [12, 13]. Because full-length recombinant OspC is difficult to express in *E*. *coli*, a non-lipidated version of OspC was constructed by eliminating the signal sequence (amino acid residues 1–18) and a high yield of expression was achieved [13–15]. Nevertheless, non-lipidated OspC is only a weak immunogen, stressing the need for strong adjuvant co-administration [15, 16].

Liposomes can incorporate antigen and/or immunomodulatory molecules and can be employed as biocompatible particulate vaccine carriers [17]. Antigenic or adjuvant molecules can also be attached by non-covalent interactions such as metallochelation, a process in which recombinant antigenic proteins in particular with 4 to 6 histidine amino acid residue extensions (His-tags) are able to chelate with divalent metal ions that are complexed to liposome membrane surfaces via metal complexes anchored to those same liposomes surfaces by lipidic tails (e.g., DOGS-NTA-Ni). Proteoliposomes may be further modified to carry adjuvants such as monophosphoryl lipid A, muramyl dipeptide (MDP) and other non-pyrogenic analogues such as Nor-AbuMDP or Nor-AbuGMDP [18–21]. Although the His-tag is a short and poorly immunogenic sequence, its attachment to a recombinant protein might mask immunologically important epitopes and reduce the potential immune response towards the target antigen, a potential impact that requires experimental evaluation [22].

In this study, we compared the immune response of experimental mice after intradermal immunization with rOspC proteoliposomes formulated with synthetic lipophilic molecular adjuvants MPLA or MT06, with rOspC formulated with a Montanide PET GEL A dispersion, with rOspC formulated with alum, or with free rOspC variants formulated without adjuvant and differing only in the N' or C' terminal attachment of a His-tag.

## Material and Methods

### General

Egg phosphatidylcholine (purity of 99%), 1,2-Dioleoyl-sn-Glycero-3-Phosphoethanolamine-N-(Lissamine Rhodamine B Sulfonyl) (LisR), 1,2-Dioleoyl-sn-Glycero-3-([N(5-Amino-1-Carboxypentyl)iminodiAcetic Acid]Succinyl)(Nickel Salt) (DOGS-NTA-Ni,) lipid were purchased from Avanti Polar Lipids (Birmingham, AL). DIO18 was purchased from Molecular Probes (Invitrogen, Carlsbad, CA). 20 nm membrane filter Anotop 10 and 0.2 μm Anotop 10 LC were purchased from Whatman (Maidstone, UK). All other chemicals, unless specially specified, were from Sigma (St. Louis, MO).

### Synthetic nor-AbuMDP analogue

**Fig 1:**
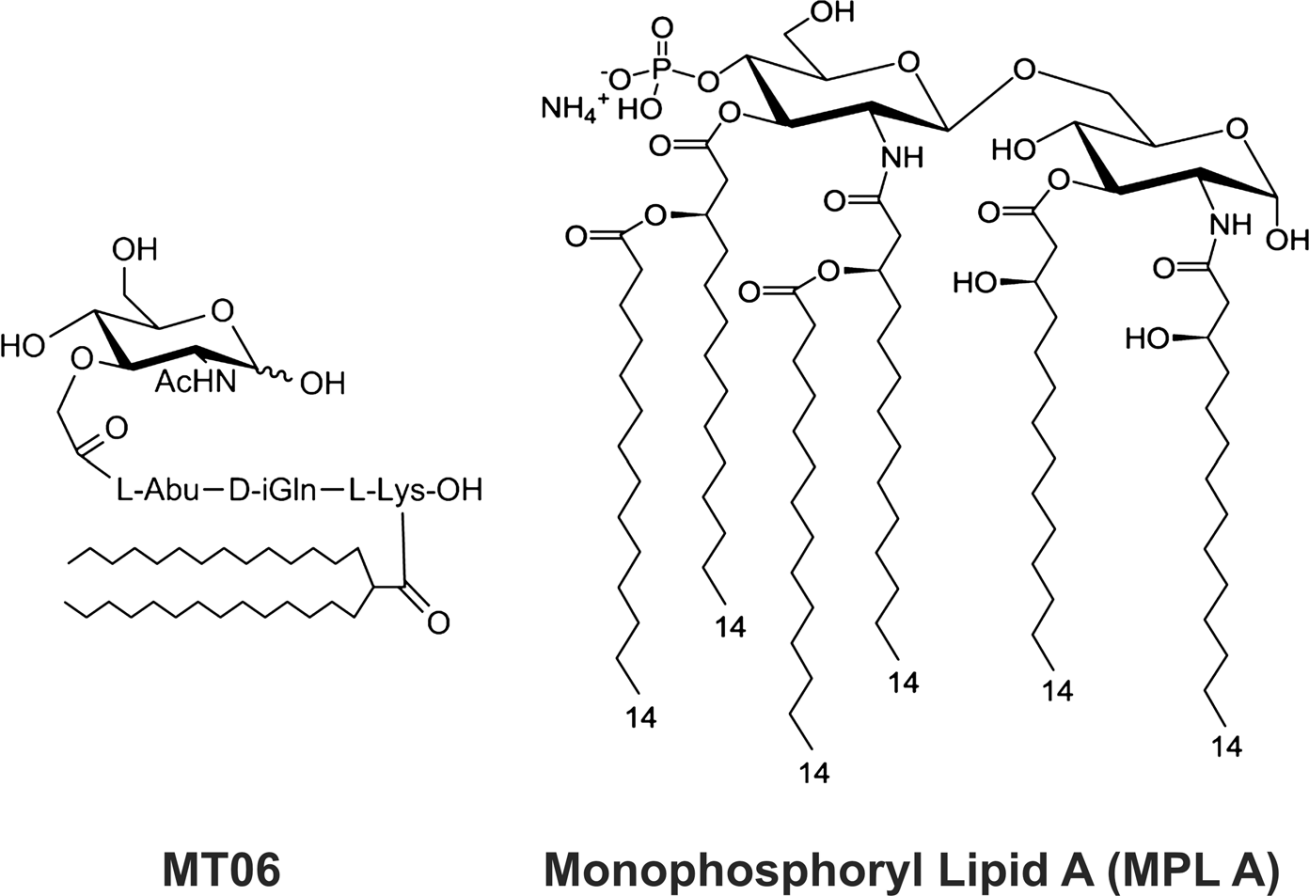
Synthetic molecular adjuvants MT06 and MPLA. Left panel shows MT06, a non-pyrogenic lipophilic derivative of muramyl dipeptide (MDP) analogue—Nor-AbuMDP. The right panel shows monophosphoryl lipid A, a derivative of lipopolysaccharide from *S*. *minnesota*.

The synthetic nor-AbuMDP analogue adjuvant, MT06. was prepared as described by Ledvina and coworkers [23] (**Fig 1**). This non-pyrogenic analogue adjuvant was selected for this study from a series of such nor-AbuMDP analogues. Monophosphoryl lipid A (MPLA) was purchased from Sigma (St. Louis, MO) (**Fig 1**). Both Montanide PET GEL A (Seppic, Paris, France) and Alum (InvivoGene, San Diego, CA) were used as standard adjuvants for comparison.

### Preparation and purification of rOspC

*B*. *burgdorferi s*. *s*. OspC cDNA (corresponding to GenBank Acc. No. EF537426), lacking the first 54 nucleotides, coding for a lipidation signal, was isolated originally from *Borrelia burgdorferi s*. *s*. B31 plasmid DNA preparation, obtained using a Qiagen plasmid purification kit (Qiagen, Hilden, Germany) according to the manufacturer's recommended modification for *Borrelia* plasmid DNA, by RT-PCR.

The PCR product of the reaction performed with downstream adapter primer (CACCATGTGTAATAATTCAGGGAAAGATGGG) und upstream primer (AGGTTTTTTTGGACTTTCTGCC) and Phusion DNA polymerase (New England BioLabs, Ipswich, MA) was cloned into *E*. *coli* expression plasmids pET101 and pET200 (Invitrogen) in order enable the expression of non-lipidated rOspC fusion proteins with a V5-tag and a C' terminal His-tag or an Xpress-tag with N' terminal His-tag. N' and C' terminal His-tag rOspC variants were purified under native conditions using the Ni-NTA agarose (Qiagen) as described earlier [15] with modified lysis buffer: (50 mM Tris; 300 mM NaCl; 10 mM Imidazole; 1 mg/ml hen egg white lysozyme; 0,1% Triton X-100; protease inhibitors: 0.2 mM PMSF; 0.4 μg/ml Leupeptin; 0.5 μg/ml Aprotinin; pH8.0). Subsequently, the proteins were dialyzed against Tris-HCl storage buffer (50 mM Tris, 150 mM NaCl, pH7.5).

### Endotoxin removal

Lipopolysaccharide (endotoxin, LPS) was removed by repeated phase extraction procedure using detergent Triton X-114 as described earlier [15], until the endotoxin level in every rOspC samples was below 2.5 EU/mg. The endotoxin concentration was measured by the gel-clot assay using Limulus Amebocyte Lysate (Associates of Cape Cod, USA).

### Characterization of rOspC by SDS-PAGE and MALDI-TOF MS

The purity of rOspC variants was analyzed using 12% T/3% C SDS-PAGE followed by staining with Coomassie Brilliant Blue (CBB) G-250. Protein identity was confirmed by peptide mass fingerprinting of SDS-PAGE-resolved samples on a Microflex LRF20 MALDI-TOF (matrix-assisted laser desorption/ionization time-of-flight) mass spectrometer (Bruker Daltonik, Bremen, Germany) as described previously [24]. In addition, the digests of the rOspC proteins were subjected to nanoflow liquid chromatography coupled with MALDI-TOF/TOF mass spectrometry (MS) and tandem mass spectrometry (MS/MS) analyses performed using an ultrafleXtreme mass spectrometer by Bruker Daltonik [25]. Proteins were then identified by peptide sequence comparisons with experimental peptide mass lists or MS/MS data, respectively, found in the NCBInr protein sequence database (September 2015) using program Mascot, version 2.2.07 (Matrix Science, London, UK).

### Preparation of rOspC-based immunization formulations

Each vaccine dose contained 20 μg of non-lipidated C' or N' terminal His-tag rOspC. Proteoliposome formulations were prepared by adding 20 μg of rOspC antigen to 300 μg of preformed EPC/POPG/DOGS-NTA-Ni/adjuvant metallochelation liposomes (71/19/5/5 mol%) (per one immunization dose), prepared by the "hydration of a lipid film" method followed by extrusion through 200 nm polycarbonate filters (Mini-Extruder, Avanti Polar Lipids, Alabaster, AL) [18, 19]. The rOspC was added to the preformed metallochelation liposomes formulated with either MT06 or MPLA as adjuvant. In addition, other soluble rOspC/adjuvant samples were prepared by mixing rOspC with alum or with Montanide PET GEL A. The composition of particular formulations is specified (**Table 1)**. As a naïve control, we used sera from mice immunized with recombinant His-tag p24-Hsp70 protein [26].

**Table 1:** Doses and vaccine formulations.

Groups of 5 mice	Composition per one dose (50 μl)					
	rOspC[μg]	Liposome[μg]	AlOH[%]	PET GEL[%]	MPLA[μg]	MT06[μg]
N' or C' rOspC	**20**	**-**	**-**	**-**	**-**	**-**
N' or C' rOspC + alum	**20**	**-**	**25**	**-**	**-**	**-**
N' or C' rOspC + PET GEL A	**20**	**-**	**-**	**5**	**-**	**-**
N' or C' rOspC + MT06 proteoliposome	**20**	**300**	**-**	**-**	**-**	**21.4**
N' or C' rOspC + MPLA proteoliposome	**20**	**300**	**-**	**-**	**32.6**	**-**
Control hsp70-p24	**-**	**-**	**-**	**-**	**-**	**-**

### Immunization of experimental mice

All experiments were performed on 6- to 8-week old female BALB/c mice purchased from Biotest (Konarovice, Czech Republic). All animals were free of known pathogens and were kept in a climate-controlled environment with 12 h light-dark-cycle, and were provided with pellet food and water *ad libitum*. The vaccination experiments were approved by the Ethics Committee of the Faculty of Medicine and Dentistry (Palacky University in Olomouc), and the Ministry of Education, Youth and Sport, Czech Republic. The animals were monitored daily by a veterinarian for their behavior, psychic activity, discharge from natural body foramens, nutrition and hydratation status, skin status and eventual lesions. The protocol for early/humane endpoints in cases where animals became severely or irreversibly ill prior to the experimental endpoint was implemented according to the animal facility rules, for recognition and assessment of pain, distress, and suffering, stating that the animals that are moribund or in a state of impending death should be immediately euthanized. The composition of doses is shown (**Table 1)**. Because all tested adjuvants were earlier confirmed as safe and not inducing severe adverse side effects [15, 19] we opted for an intradermal (i.d.) route of vaccines administration in order to obtain intense immune responses. All mice were immunized twice with particular rOspC formulations (priming followed by boosting 14 days) by *i*.*d*. administration using a G29 needle into fur clipped and 70% ethanol disinfected skin of the ventral abdomen. The needle was inserted, bevel up, nearly parallel to the plane of the skin for at least 8 mm. The solution was slowly injected. The formation of a hard bleb was taken as a sign of successful administration of the vaccine. Blood samples (130 μl per mouse) were obtained using tail vein sample collection approach. During the experiment, none mouse died nor exhibited clinical signs of pain, distress, or suffering. After termination of the experiment, the animals were euthanized under Katemine/Xylazine anesthesia by cervical dislocation.

### OspC-specific serum antibody levels determination by ELISA

All assays were performed in duplicates. For all ELISA, rOspC was devoid of all tags using recombinant enterokinase as described recently [26]. ELISA assays were performed as described [26]. In brief, ELISA wells were coated with 100 ng/well of non-tag rOspC. OspC-specific IgG+IgM+IgA (IgP), IgG1, IgG2a levels were then measured. The results were expressed as the specific antibodies O.D. determined at linear proportion of sera dilution determined in preliminary experiment using Genesis Lite Software (Version 3.03, Life Sciences, Basingstoke, UK).

### Transmission electron microscopy and immunogold labeling of N' and C' terminal His-tag rOspC proteoliposomes

The liposome structures were determined using transmission electron microscope (EM Philips 208 S, MORGAGNI software, FEI, CZ). Non-liposome bound rOspC was separated from the proteoliposomes by Superose 6 gel permeation chromatography. The proteoliposome fractions were then concentrated using ultrafiltration centrifugation tube (cut off 30 kDa) and incubated with murine OspC-positive or -naȉve sera diluted 1:50 for 1 h at 37°C. 10-nm colloidal gold–protein A conjugate was added. After 12 h incubation, the proteoliposomes were negatively stained by 2% (w/w) ammonium molybdate (pH 6.8).

### Determination of secondary structure and thermal stability of His-tag rOspC proteins by circular dichroism

Circular dichroism (CD) spectra were obtained at room temperature (22°C) using a Chirascan CD spectrometer (Applied Photophysics, United Kingdom). Data were collected from 185 to 260 nm at 100 nm/min, 1 s response time, and 2 nm bandwidth using a 0.1 cm quartz cuvette containing the rOspC in 50 mM potassium phosphate buffer, pH 7.5. Each spectrum shown is the average of five individual scans and was corrected for absorbance of the buffer. CD data were expressed as the mean residue ellipticity. The proportion of certain secondary structure was calculated from the measured spectra using CDSSTR, Selcon3, and CONTIN methods using DichroWeb server. Thermal unfolding of rOspC was followed by monitoring the ellipticity at 195 and 222 nm over the temperature range of 20 to 80°C, resolution of 0.1°C, at a heating rate of 1°C/min. Recorded thermal denaturation curves were roughly normalized to represent signal changes between approximately 1 and 0 and fitted to sigmoidal curves using software Origin 6.1 (OriginLab, Massachusetts, USA). The melting temperature (Tm) was evaluated as a midpoint of the normalized thermal transition.

### Determination of secondary structure of His-tag rOspC proteins by Fourier transform infrared (FTIR) spectrometry

The infrared spectra of individual rOspC were obtained using a FTIR spectrometer TENSOR 27 (Bruker Optics, Germany) with an AquaSpec flow-through transmission cell and operating at 4 cm^-1^ spectral resolution. The protein concentration in 50 mM potassium phosphate buffer, pH 7.5, was 1 mg/ml. The same buffer was used for the background measurements. The secondary structure was determined by analyzing the amide-I band (1700–1600 cm^−1^) via a multivariant pattern recognition method based on a FTIR spectra library provided by the manufacturer.

### Determination of thermal stability by DSC

Differential scanning calorimetry (DSC) measurements were performed using DSC 6100 Nano-Differential Scanning Calorimeter II (Setaram, Caluire, France) with a cell volume of 0.3 ml. Prior the injection, the samples in Dulbecco’s PBS without calcium chloride and magnesium chloride were extensively degassed. The protein concentration of C' and N' terminal His-tag rOspC was 6.9 and 6.2 mg/ml, respectively. Scans ran from 10 to 85°C at a scan rate of 1°C per minute. The reference-baseline was obtained by buffer vs. buffer scan and subtracted from the data measurement. The apparent molar heat capacity and excess molar heat capacity curves were obtained from the calorimetric profiles corrected by baseline subtraction.

### Determination of thermal stability by DLS

Thermal stability of C' and N' terminal His-tag rOspC proteins was determined also by dynamic light scattering (DLS). The hydrodynamic diameters of rOspC were monitored over the temperature range of 25–55°C at the temperature gradient of 1°C/ min. The concentration of rOspC proteins in Dulbecco’s PBS without calcium chloride and magnesium chloride was 1 mg/ml.

### Statistical analyses

Statistical significance was determined by analysis of variance (ANOVA) followed by Dunnett's Multiple Comparison Test or Student-Newman-Keuls (SNK) Multiple Comparison Test. All statistical analyses were performed using GraphPad Prism 5 software (GraphPad Software Inc, CA, USA).

## Results

### Purification and identification rOspC antigens

**Fig 2:**
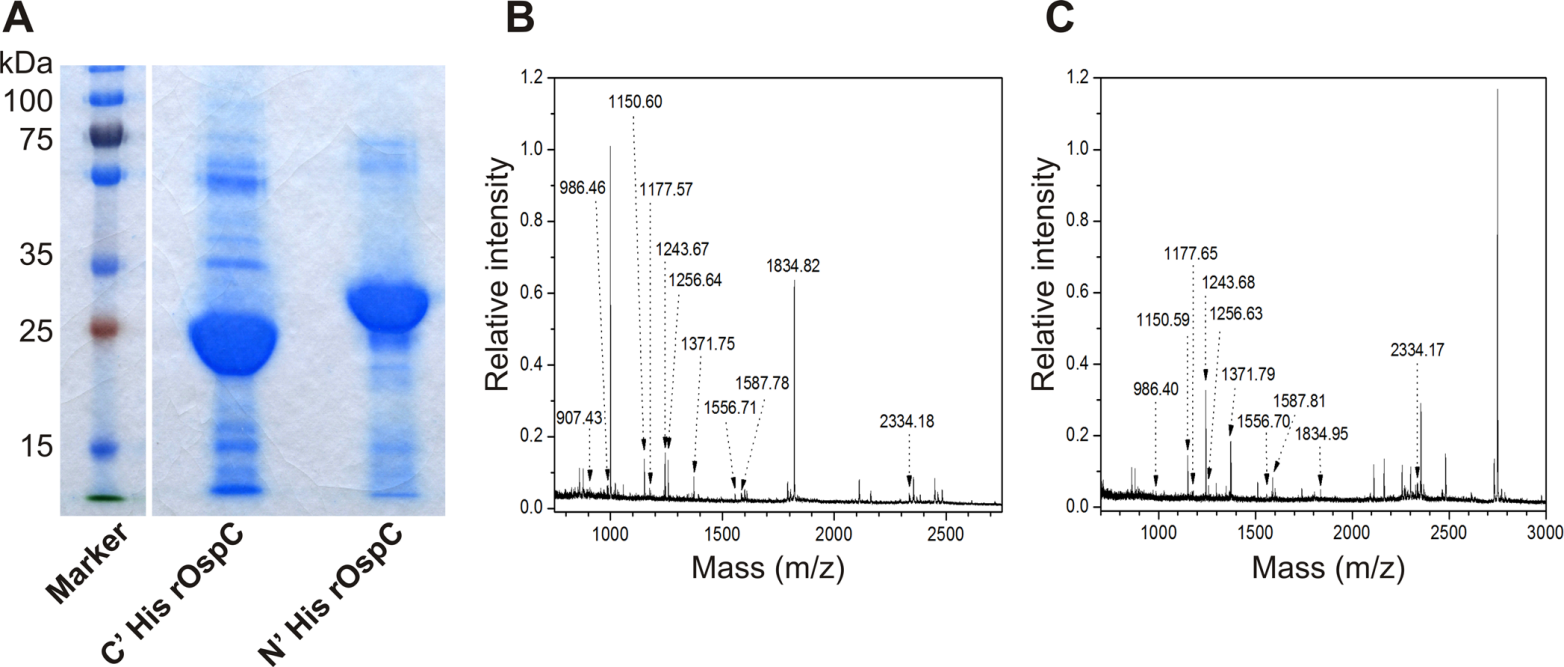
SDS-PAGE and MS analyses of recombinant N' and C' terminal His-tag rOspC. **A)** Recombinant N' and C' terminal His-tag rOspC proteins were separated using SDS-PAGE and stained by CBB G-250. The differences in mobility of both rOspC variants are caused by the length of labeling tags and spacers and correspond to theoretical prediction based on cDNA sequences: 23.96 kDa for C' and 27.12 for N' terminal His-tag rOspC. **B, C**) MALDI-TOF peptide mass fingerprinting of rOspC proteins. All spectra were acquired using CHCA matrix on Microflex LRF20 MALDI-TOF mass spectrometer. Panel **B**) refers to C' terminal His-tag rOspC and panel **C**) N' terminal His-tag rOspC.

Two rOspC variants were expressed in *E*. *coli*, reaching a yield of approximately 30 mg of rOspC per 1 L of culture. Due to different cloning strategies, both rOspC variants differed in theoretical molecular weight: 27.12 kDa for N' terminal His-tag rOspC and 23.96 kDa for C' terminal His-tag rOspC (ProtParam tool; www.expasy.ch). These molecular weights were confirmed experimentally by SDS-PAGE with CBB staining (**Fig 2A**). The purity of both rOspC variants was confirmed as higher than 93% by comparing the density of rOspC bands with all other visible bands on gel (using ImageJ 1.41a software).

The identities of both rOspC variants were further confirmed by peptide mass fingerprinting using MALDI-TOF MS, wherein the dominant protein bands (indicated by arrows **Fig 2A**) were sequence matched with the known OspC protein sequence (NCBInr gi|374718430). Sequence coverage was 63% (13 peptides) for both N' and C' terminal His-tag rOspC. The corresponding probability-based MOWSE scores were 157 and 162, respectively. Examples of peptide MS spectra for both rOspC variants are provided in **Fig 2B and 2C**. In addition, nanoLC-MALDI-TOF/TOF MS and MS/MS were used for peptide-matching with respect to two other known OspC sequences: gi|68161620 for N' terminal His-tag rOspC (25 peptides, sequence coverage—75.7%, score—2743) and gi|1695212 for C' terminal His-tag rOspC (14 peptides, sequence coverage—77.6%, score—1825). The two recombinant proteins were distinguished by means of specific peptides found in respective peptide digests, such as *m/z* 2255.9 (DLYDDDDKDHPFTCNNSGK) for N' terminal His-tag rOspC.

### Immunization of mice with rOspC antigens

**Fig 3:**
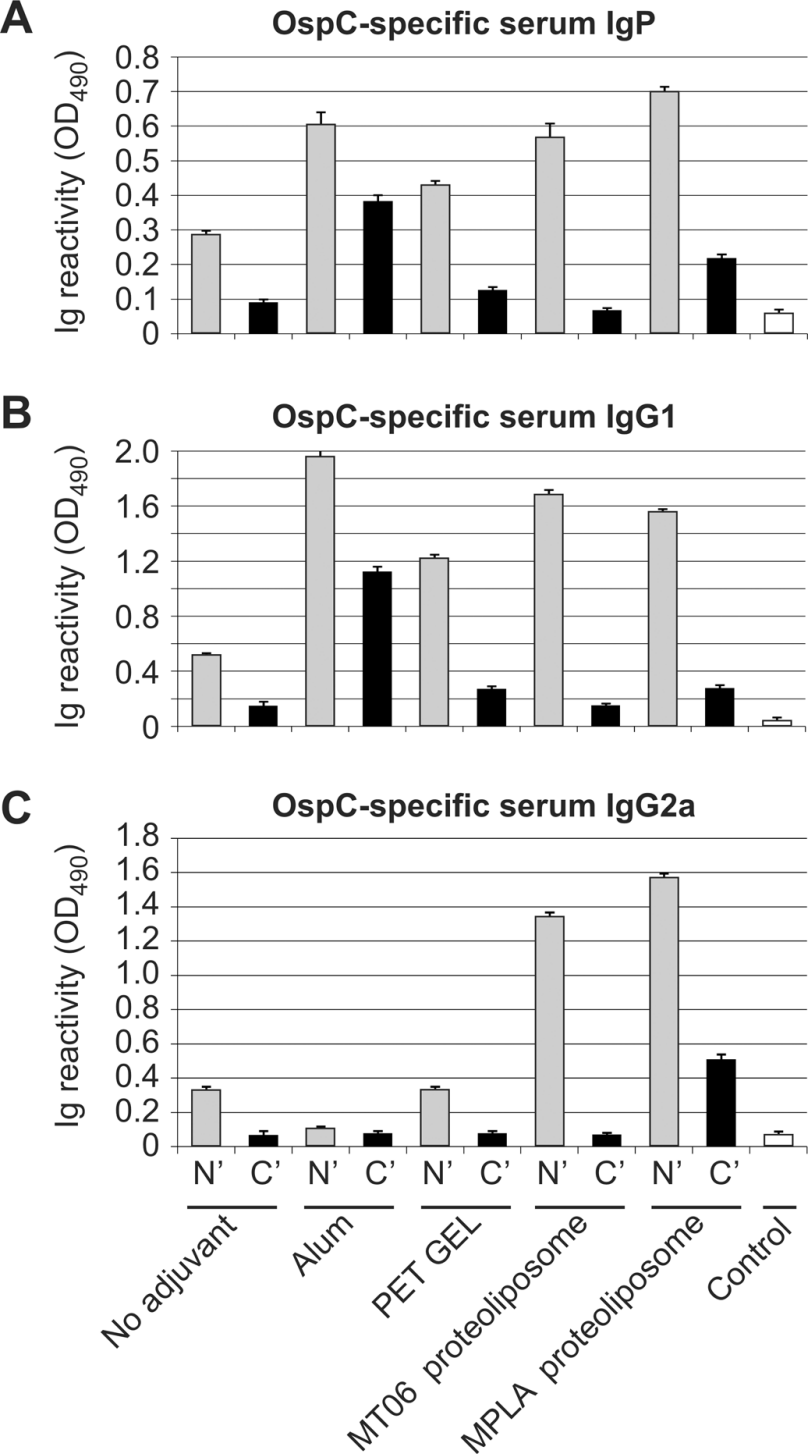
Determination of rOspC-specific antibodies in sera of immunized mice by ELISA. Mice (5 per group) were immunized twice in 14 days interval by i.d. administration of various N' and C' His-tagged rOspC formulations as specified in **Table 1**. Pooled sera from mice in each group were used for ELISA analysis of OspC-specific antibodies. Control sera were obtained from mice immunized by recombinant His-tagged p24-Hsp70 without adjuvants [26]. ELISA plates were coated with 0.1 μg of non-tagged rOspC, incubated with serially diluted pooled sera obtained 14 days after second immunization and developed with HRP-conjugated anti-mouse **A**) IgG+IgM+IgA (IgP), **B**) IgG1, and **C**) IgG2a, developed with orthophenylene diamine. The absorbance was read at 490 nm. The response of mice immunized by N' His-tagged rOspC are in gray, and C' His-tagged rOspC are in black. The results are expressed as mean absorbance (O.D.) at optimal sera dilutions (1:4000 for IgP and IgG2a; 1:8000 for IgG1) corresponding to lineal portion of titration curve +/- SD. The expressed values correspond to means from three independent immunization experiments.

Using an ELISA approach, we identified that immunizations with soluble rOspC without any adjuvant elicited significant increases in the levels of OspC-specific antibodies response in total Ig isotypes (IgP), but only when N' terminal His-tag rOspC was used whereas C' terminal His-tag rOspC was significantly less effective (SNK test, P<0.001) (**Fig 3A**). Similarly, even with adjuvants present, all groups of mice immunized with C' terminal His-tag rOspC exhibited substantially lower antibody responses in comparison to groups immunized with N' terminal His-tag rOspC (SNK test, P<0.001) (**Fig 3A**). Otherwise, in terms of the antigen-adjuvant combinations tested, Montanide PET GEL A/His-tag rOspC combinations appeared the weakest antigen-adjuvant combinations tested (SNK test, P<0.001), whilst the N' terminal His-tag rOspC proteoliposomes combination formulated with MPLA was the most effective antigen-adjuvant combination tested (SNK test, P<0.001). The N' terminal His-tag rOspC proteoliposomes combination formulated with MT06 was able to induce equivalent levels of OspC-specific antibodies to the N' terminal His-tag rOspC/alum antigen-adjuvant combination (SNK test, P<0.001) (**Fig 3A**). Furthermore, alum was found to be the most effective adjuvant to help induce antibody responses with C' terminal His-tag rOspC immunization (SNK test, P<0.001).

In further experiments, we compared the stimulated levels of two specific IgG isotype antibodies following immunization with N' terminal His-tag rOspC/adjuvant combinations. The IgG isotype antibodies analyzed were IgG1 representing the Th2 type and IgG2a representing the Th1 type of immune response. Quite clearly, the presence of alum elicited the highest levels of OspC-specific IgG1 antibody production but only very weak corresponding IgG2a antibody production levels (SNK test, P<0.001) (**Fig 3B and 3C**). Similarly, the presence of Montanide PET GEL A also resulted in respectable levels of OspC-specific IgG1 antibody production but also only weak corresponding IgG2a antibody production. Conversely, N' terminal His-tag rOspC proteoliposomal formulations, using either MPLA or MT06 as adjuvants, induced strong immune responses involving IgG1 and IgG2a isotypes (**Fig 3B and 3C**) and the IgG2a responses were substantially greater than the corresponding IgG2a responses seen when alum or Montanide PET GEL A were used as adjuvants, or when free N' terminal His-tag rOspC was administered without adjuvant (SNK test, P<0.001) (**Fig 3C**). Otherwise, when C' terminal His-tag rOspC was tested, only proteoliposomes with added MPLA induced detectable increases in OspC-specific IgG2a antibodies (SNK test, P<0.001), while alum helped promote a surprisingly effective C' terminal His-tag rOspC mediated induction of IgG1 responses (**Fig 3B**) (SNK test, P<0.001). Of note, administration of rOspC proteoliposomes was associated with lowest, practically undetectable irritation at the application site (data not shown).

### Immunogold TEM analyses of rOspC proteoliposomes

**Fig 4:**
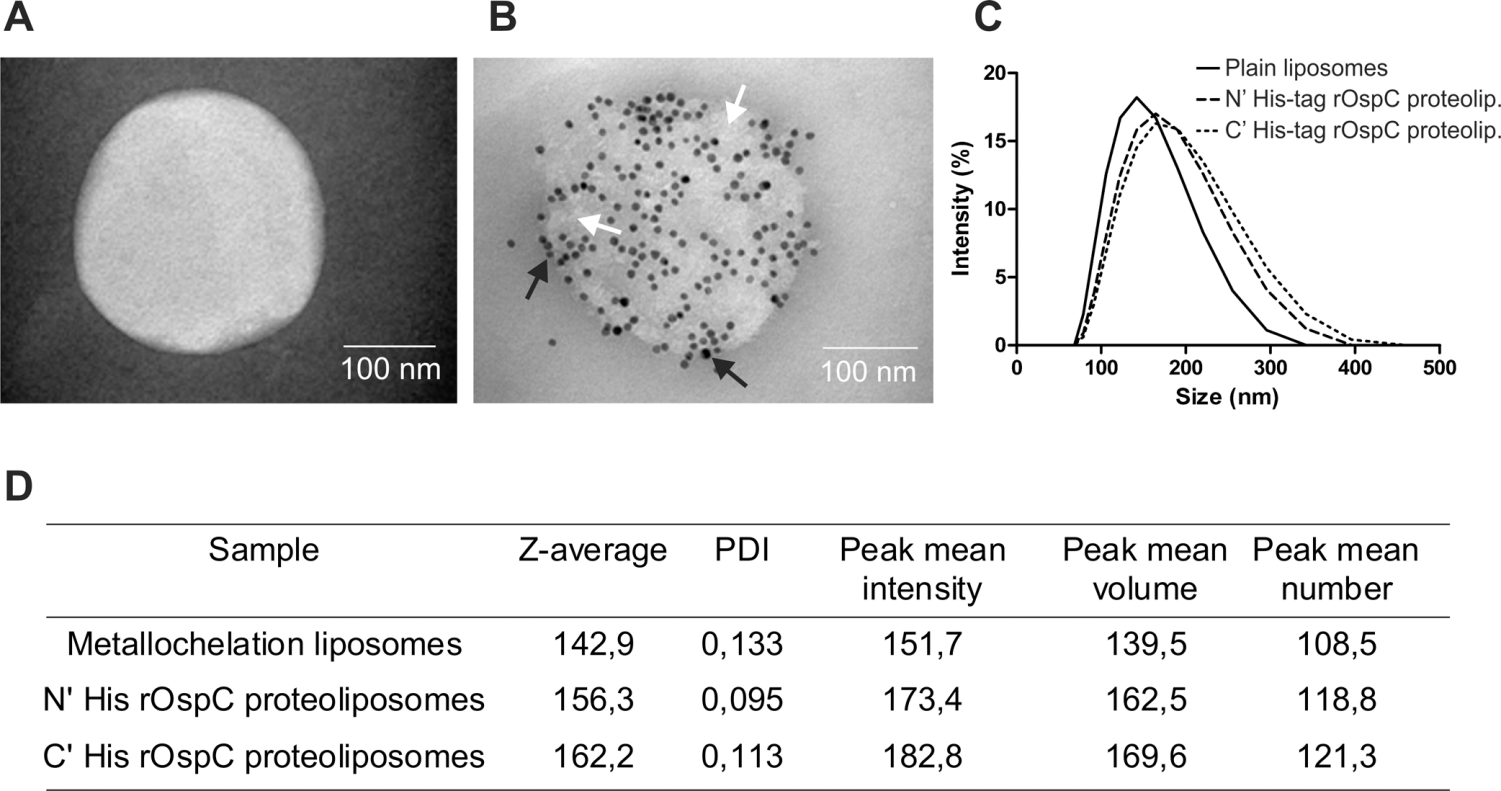
Characterization of metallochelating N' and C´ terminal His-tag rOspC proteoliposomes by immuno EM and DLS. The EM picture of **A)** plain nanoliposome, **B**) metallochelation nanoliposome with surface bound N´ terminal His-tag rOspC proteins. Metallochelation liposomes were incubated with N' and C' terminal His-tag rOspC proteins followed by incubation with pooled sera from rOspC-immunized mice (1:50 dilution) followed by addition of protein A-labeled 10-nm colloidal gold particles. After 12-h, the proteoliposomes were negatively stained by ammonium molybdate and observed using Philips Morgagni transmission EM. Black dots represent immunogold particles on rOspC proteins bound to the liposome surface (black arrows). rOspC protein molecules (white dots) forms chains on proteoliposome surfaces (white arrows). **C)** The increase of hydrodynamic radius after binding of rOspC proteins was measured by DLS. **D)** Tabular data characterizing the size of plane and rOspC proteoliposomes in detail.

As the N' terminal His-tag rOspC proteoliposomes with MT06 or MPLA exhibited highest efficacy in inducing the OspC-specific IgG2a antibodies, these proteoliposomes were characterized in more detail using TEM (**Fig 4).** Gold nanoparticles labeling of rOspC gave rise to the appearance of short rOspC chains distributed on the liposome surface. Similar pattern was noted for C' terminal His-tag rOspC (*data not shown*). When OspC-naïve mouse sera were used for labeling, no positivity of immunogold was observed (*data not shown*).

### Analyses of the secondary structure of rOspC proteins

**Fig 5:**
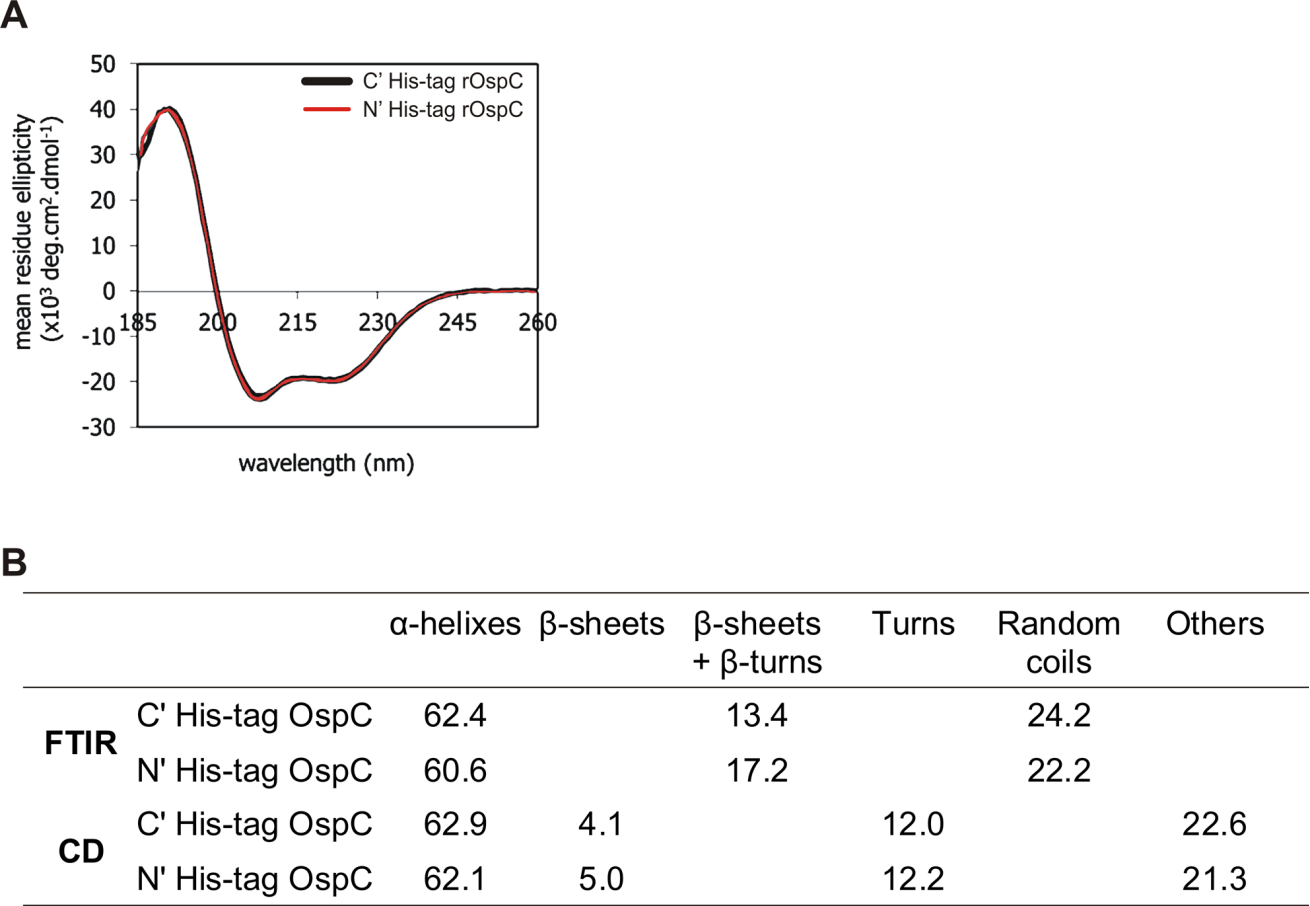
CD spectra of rOspC proteins. Secondary structure of N' and C' terminal His-tag rOspC proteins determined by CD and FTIR. **A**) CD and FTIR spectra were obtained at room temperature (22°C) using a Chirascan CD spectrometer and Tensor 27 FTIR, respectively. **B)** Comparison of secondary structures of rOspC proteins obtained by calculations based on FTIR and CD measurements.

Both rOspC proteins exhibited CD spectra with two sharp negative maxima at 222 and 208 nm and one highly intense positive maximum at 195 nm characteristic of α-helical structure, suggesting that the position of His tag has no detectable effect on the secondary structure (**Fig 5A**). The analysis of the spectra revealed that the secondary structure is predominantly helical. The predicted α-helical content of both measured rOspC proteins was about 62% (**Fig 5B**) whereas the β-sheet content was only about 5%. The secondary structure determined by FTIR was in good correlation with the CD prediction (**Fig 5B**). Both techniques confirmed that the position of the His-ag at either terminus did not result in significantly alteration to rOspC protein secondary structure.

### Assessment of thermostability of rOspC proteins

**Fig 6:**
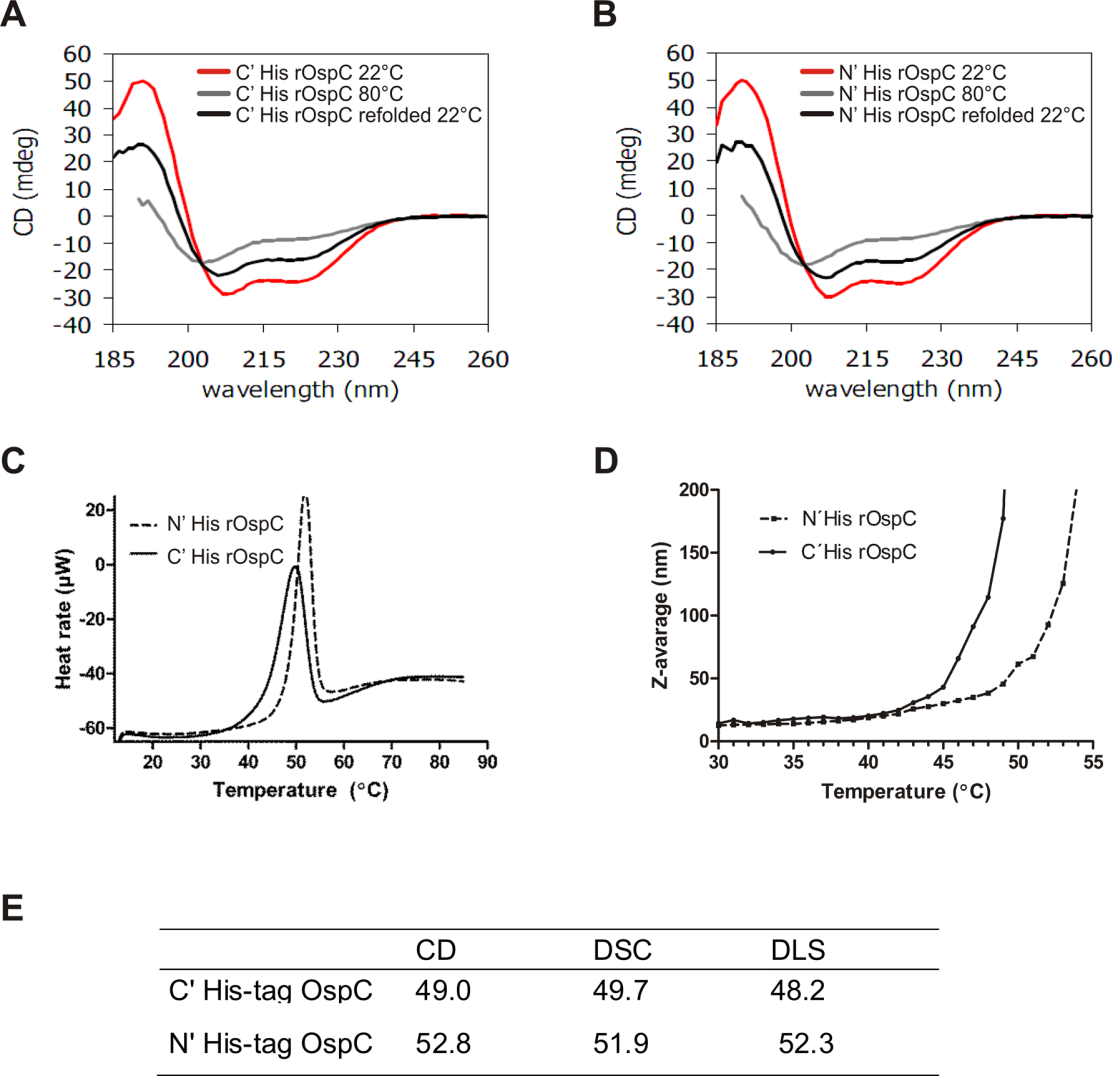
Determination of thermal stability of His-tag rOspC proteins by DLS, DSC and circular dichroism. **A, B**) rOspC ellipticity induced by temperature changes monitored by circular dichroism. CD spectra were obtained at room temperature (22°C) using a Chirascan CD spectrometer. Data were collected from 185 to 260 nm at 100 nm/min, 1 s response time, and 2 nm bandwidth using a 0.1 cm quartz cuvette. Thermal unfolding of rOspC proteins was followed by monitoring the ellipticity at 195 and 222 nm over the temperature range of 20 to 80°C, with a resolution of 0.1°C, at a heating rate of 1°C/min. **C**) Determination of thermal stability by measurement transition midpoints of rOspC proteins using DSC. Scans ran from 10 to 85°C at the scan rate of 1°C per minute. **D**) Thermal stability of rOspC determined by DLS. The hydrodynamic diameter of the proteins was monitored over the temperature range of 25–55°C. The lines are created from hydrodynamic radius measurements at the temperature gradient of 1°C/ min. **E**) Transition temperatures (°C) of of N' and C' terminal His-tag rOspC calculated from DLS, DSC, and FTIR measurements.

Thermally induced denaturation of rOspC proteins was followed to explore their structural stability and the reversibility of their refolding behavior (**Fig 6**). Thermal unfolding of both rOspC proteins was monitored by CD spectroscopy (**Fig 6A and 6B**), DSC (**Fig 5C**) and DLS (**Fig 6D**) as a function of temperature. Changes in CD signal induced by increased temperature increases were very similar for both rOspC proteins, and melting temperatures of 49.0 and 52.8 were calculated for C' and N' terminal His-tag rOspC respectively (**Fig 6E**). In order to determine the extent of reversibility of post thermally induced unfolding, CD spectra were measured prior to heating (22°C), at the temperature of denaturation (80°C) and again after cooling (22°C) (**Fig 6A and 6B**). Both rOspC proteins exhibited similarly irreversible thermal denaturation. The CD spectra of rOspC proteins after heating and cooling suggested that of α-helical character was retained but that this was still less than comparable levels seen in native proteins. Similar behavior was observed also when both rOspC proteins were heated to their transition midpoint at a heating rate of 1°C/min and then consequently cooled back to 22°C. The CD data were in a good agreement with data obtained by DSC and DLS. The transition midpoints of C' and N' terminal His-tag rOspC as determined by DSC were 49.7 and 51.9°C, respectively (**Fig 6C**). The cooling curves confirmed the irreversible unfolding of both rOspC proteins. Also the unfolding curves obtained by DLS demonstrated a dramatic Z-average increase at 48.2 and 52.3°C for C' and N' terminal His-tag rOspC, respectively (**Fig 6D**). Hence the results obtained by the three techniques were in a good agreement as regards protein melting points and the irreversibility of thermal protein unfolding. They show that N' terminal His-tag rOspC is more thermally stable than C' terminal His-tag rOspC.

## Discussion

OspC antigen represents one of the most promising candidate antigens for Lyme disease-preventing vaccine since it is expressed by *Borrelia* during transmission from tick to vertebrate host and during an early phase of vertebrate infection, thus providing the window for targeting the *Borrelia* by OspC-specific antibody-mediated opsonization or complement-activation.

In this study we compared immunogenicity of two rOspC variants that differed in possessing a His-tag at either N' or C' terminus. Such His-tag used both to assist purification and to assist the anchoring of rOspC proteins onto the surface of metallochelation liposome. Given this, the necessity of comparing and contrasting these two protein variants arose from published reports showing that the N' versus C' position of His-tag can adversely affect conformation [27], and/or impair protein immunogenicity for a variety of potential reasons [28].

On our case, in comparing N' versus C' terminal His-tag rOspC administered as soluble proteins with or without adjuvant, all N' terminal His-tag rOspC formulations exhibited substantially higher immunogenicity compared with all C' terminal His-tag rOspC variant formulations. Therefore, when protein secondary structures of both rOspC variants were compared using CD and FTIR spectroscopy, no substantial differences were observed in the content of α helix, β sheet/β turn and random coil structures. On the other hand, thermal stability analyses performed by CD, DSC, and DLS did suggest that N' terminal His-tag rOspC was slightly more thermally stable which could contribute at least partially to preserving rOspC epitopes *in vivo* after rOspC administration to mice. Still, in our case, changes in conformation as well as in thermal stability would only seem to play a minor role in modifying N' terminal His-tag rOspC and C' terminal His-tag rOspC epitope structures and their availability for immune recognition. Rather the best explanation for the distinctly different immunogenicities of both rOspC variants is that C' terminal His-tag sterically occludes access to critical adjacent C' terminal epitopes in a way that the N’ terminal His-tag does not. This suggestion is corroborated by observations made using human early Lyme disease sera wherein dominant immunoprotective OspC epitopes were found to be located close to the conserved C' terminus [10, 11, 29]. On the other hand, the proximity of the N' and C' termini of OspC in crystallographic data [30], might suggest that an N' terminal His-tag could also have a steric impact on access to C' terminal epitopes as well although the immunostimulatory data presented here would suggest that this potential effect is much less of a problem than might be expected. Also our data (**Fig 3**) do give some grounds for suggesting that the inhibitory effect of a C' terminal His-tag can be overcome at least in part with a well selected adjuvant such as alum and to some extent MPLA, both of which appear able to boost immune responses otherwise interfered with by steric occlusion, as described above. Such differential immunostimulatory behavior between rOspC variants may be a general problem in different species with their different immune systems [29]. Still, further experimental work would clearly be needed to verify such a suggestion.

Other alternative *Borrelia* antigens for vaccination purposes are OspA, OspB, DbpA and BBbk32 proteins. In 1998, the US FDA approved a recombinant OspA (rOspA)-based vaccine known as Lymerix that acts to prevent *Borrelia* transmission. Nevertheless, due to media-evoked concerns about potential autoimmune reactions of OspA, linked to its partial homology with the human integrin LFA-1 (CD11a/CD18), this recombinant vaccine has been withdrawn from the market since 2002. Various recombinant proteins derived from identified protective *Borrelia* antigens have also been expressed as fusion proteins together with various tags such as the glutathione S-transferase (GST) tag [31], the maltose-binding protein tag [32], or His-tag [33]. Interestingly, Bockenstedt et al., 1996 reported that the addition of a His-tag to a C' terminal fragment of OspA substantially enhanced immunogenicity in comparison to the situation with a GST-tag [34], suggesting that inclusion of the GST tag had induced significant conformational changes in the associated OspA antigen to impair immunogenicity [33]. When Koide et al, 2005 introduced an N' terminal His-tag to an OspA C' terminal fragment, the resulting recombinant protein became stabilized by residues promoting hydrophobic interactions and hence the vaccination efficacy of the redesigned OspA fragment was found to be equivalent to full-length OspA protein [35]. OspA is not necessarily an ideal antigen for anti-Borrelia vaccination given the diversity of known OspA serotypes. Still Comstedt et al, 2014 has reported that OspA dimers can be used for immunization of mice following the protein engineering of disulfide bridges to stabilize the C'-terminal region of various His-tag OspA fragments [36]. On the other hand, Earnhart et al, has reported on various N'-terminal His-tag chimeric OspC polyepitopes comprising the OspC C'-terminal region composed of loop 5 and α-helix 5 from invasive *Borrelia* isolates. Studies with these polyepitopes demonstrated that the immunogenicity of individual epitopes depends on individual epitope positions in sequence as well as the inclusion of the highly conserved C'-terminal OspC fragment [37].

In terms of commercialization potential and reduction to practice, large-scale production of rOspC is hindered by the low yield of lipidized rOsp antigens. Therefore we opted to use rOspC without a lipidation signal (expressing at ~ 28 mg per 1 L of bacterial culture) [15] but with a reduced level of immunogenicity compared to other *Borrelia* Osp antigens [15, 38]. OspC-specific antibody responses were then elicited in mice with either soluble rOspC or in the presence of various adjuvants such as Montanide PET GEL A and MPLA, a TLR4 ligand derived from the LPS of the Gram negative *Salmonella minnesota* R595 bacteria. In contrast to alum, that is known to promote Th2 responses, MPLA has been shown to induce preferentially Th1-biased immune responses in mice [39]. Accordingly, in our case we observed a potent immunostimulatory activity shifted towards the Th2 type of antibodies (IgG1) in response to the administration of soluble rOspC variants in conjunction with alum, in line with previous reports concerning to use of rOspC or rOspA [15, 40]. In contrast, rOspC proteoliposomes formulated with MPLA, followed by MT06, induced strongest OspC-specific Th1 antibody responses determined by the dominance of antibody IgG2a isotype [4, 5, 15, 40, 41]. The Montanide PET GEL A was generally the weakest adjuvant that was evaluated although OspC-specific Th1 antibody responses were also observed. Finally, in order to exclude the possibility that the observed differences in OspC-specific antibody responses were linked to particular rOspC sequence variants used in ELISA, we determined the reactivities of sera using the Lineblot OspC assay and found that the Lineblot OspC exhibited similar trends (data not shown).

Obviously the design of rOspC antigen used in the experiments described here does not appreciate the substantial variability of OspC confirmed from clinical isolates of *Borrelia* which would need to be taken in account in developing an actual vaccination system. Furthermore, experimental animals and humans have different pattern of epitope recognition too [5, 42–46]. Nevertheless, given the extensive use of His-tags and their potential use for efficacious affinity purification and oriented anchoring of antigenic proteins such as rOspC onto surface such as liposomes for vaccination purposes [14, 18, 19, 47] then there is a clear need to demonstrate the potentially pivotal significance to protein immunogenicity should the His-tag be located at N' or C' termini.

In conclusion, we have compared the immunogenicity of N' and C' terminal His-tag rOspC antigens in mice and identified that N' terminal His-tag rOspC induced strong OspC-specific antibody response whereas the C' terminal His-tag rOspC was found to be a much poorer immunogen. Both rOspC variants exhibited similar α-helix and β-sheet composition and were closely similar in terms of their thermal stability (until 42°C). Likewise, EM analyses of rOspC proteoliposomes identified no differences between both rOspC variants. Thus the observed differences in OspC-specific antibody responses are probably linked predominantly to direct steric hindrance of C' terminal rOspC epitopes brought about with a neighboring C' terminal His-tag. Based on our data we can conclude that a N' terminal His-tag rOspC attached by metallochelation to liposomes formulation with MPLA or MT06 adjuvant are a promising starting point for the development of Lyme disease-preventing vaccines with both veterinary and human applications.
